# Metabolic impact of residual C-peptide secretion in type 1 diabetes mellitus

**DOI:** 10.20945/2359-4292-2023-0503

**Published:** 2024-10-17

**Authors:** Valentim Lopes, Maria Eduarda Sousa, Sara Campos Lopes, Adriana De Sousa Lages

**Affiliations:** 1 Departamento de Endocrinologia ULS Braga Braga Portugal Departamento de Endocrinologia, ULS Braga, Braga, Portugal; 2 Faculdade de Medicina Universidade do Minho, Braga Portugal Faculdade de Medicina, Universidade do Minho, Braga, Portugal; 3 Faculdade de Medicina Universidade de Coimbra Coimbra Portugal Faculdade de Medicina, Universidade de Coimbra, Coimbra, Portugal

**Keywords:** C-peptide, insulin, type 1 diabetes mellitus, continuous glucose monitoring

## Abstract

**Objective:**

This study aimed to evaluate the association of detectable C-peptide levels with various continuous glucose monitoring (CGM) metrics and diabetes complications in patients with type 1 diabetes mellitus (T1DM).

**Subjects and methods:**

Retrospective, descriptive study of 112 patients with T1DM undergoing intensive insulin therapy, categorized according to fasting C-peptide level into undetectable (<0.05 ng/mL) and detectable (≥0.05 ng/mL) groups.

**Results:**

The patients’ median age at diagnosis was 22 (12-34) years and the median T1DM duration was 18.5 (12-29) years. Patients with detectable versus undetectable C-peptide levels were older (27.5 [16.5-38.5] versus 17.5 [9.8-28.8] years, respectively, p = 0.002) and had shorter disease duration (14 [9-24] versus 20 [14-32] years, respectively, p = 0.004). After adjustment for covariates (sex, disease duration, body mass index, and use of continuous subcutaneous insulin infusion), detectable C-peptide level was associated with lower time above range (TAR; aβ -11.03, p = 0.002), glucose management indicator (GMI, aβ -0.55, p = 0.024), and average glucose (aβ -14.48, p = 0.045) and HbA1c (aβ -0.41, p = 0.035) levels. Patients with detectable versus those with undetectable C-peptide level had significantly higher time in range (TIR) before (β = 7.13, p = 0.044) and after (aβ = 11.42, p = 0.001) adjustments. Detectable C-peptide level was not associated with lower time below range (TBR), coefficient of variation (CV), or prevalence of chronic microvascular and macrovascular complications.

**Conclusions:**

Persistent C-peptide secretion in patients with T1DM was associated with significantly better metabolic control reflected by different glucose metrics, namely, TIR, TAR, GMI, and HbA1c.

## INTRODUCTION

According to the International Diabetes Federation (IDF), an estimated 537 million adults worldwide ( *i.e.* , 10.5% of the world population) had diabetes mellitus in 2021 ( 1 ). In Portugal, the National Diabetes Observatory (OND) estimated the prevalence of diabetes mellitus to be 13.6% in 2018 ( 2 ).

Of all types of diabetes, the most common are type 2 (T2DM) and type 1 (T1DM) diabetes mellitus. Patients with T1DM undergo immune-mediated beta-cell destruction, with autoantibodies present in 70%-90% of the cases ( 3 , 4 ). Patients with T2DM have an initial phase of insulin resistance followed by pancreatic beta-cell dysfunction, which subsequently compromises insulin secretion ( 5 ).

Notably, T1DM is one of the most common endocrine and metabolic conditions in childhood. The T1DM pathogenesis can be divided into stages related to the presence of autoantibodies and progress from beta-cell destruction and dysglycemia to, ultimately, symptoms associated with hyperglycemia ( 6 ).

One of the ultimate goals of insulin therapy is to mimic normal insulin secretion. Intensive insulin replacement is currently the mainstream therapy for T1DM. In most people with T1DM, beta cells are destroyed after a short honeymoon period that lasts between 1 and 2 years from diagnosis. However, pancreatic beta cells may retain some functional viability decades after T1DM diagnosis ( 7 ).

C-peptide is secreted in an equimolar ratio of 1:1 for an insulin molecule, representing an indirect measure of endogenous insulin secretion. Compared to insulin, C-peptide has a longer half-life and negligible hepatic metabolization. This translates into systemic circulating levels of C-peptide being approximately five times higher than those of insulin. In clinical practice, the measurement of C-peptide levels is of great interest for the diagnosis and classification of diabetes mellitus. This method is also reliable, economically accessible, and widely available. In recent years, the usefulness of measuring C-peptide in patients with T1DM has been investigated, since its residual secretion may have clinical implications in the course of the disease, particularly in terms of therapeutic strategies during follow-up ( 8 ).

Significant residual C-peptide secretion is more frequent in adult patients and may be detected in 36% of the patients in the first 5 years and 22% of those 20 years after T1DM diagnosis ( 9 ). During the first 7 years after T1DM diagnosis, there is an abrupt drop in C-peptide values, followed by stabilization in the subsequent years ( 10 ).

Based on data from the Diabetes Control and Complications Trial (DCCT), intensive insulin therapy seems to prolong the duration of detectable C-peptide levels in people with T1DM. This preserved C-peptide secretion has been associated with decreased frequencies of severe hypoglycemia and microvascular complications, lower absolute glycated hemoglobin (HbA1c) values, and reduced insulin requirements ( 11 , 12 ). Regarding the effect of intensive insulin therapy on new glucose metrics ( *e.g.* , glycemic variability), the data are still limited but suggest lower glycemic variability in patients with preserved C-peptide secretion ( 13 ).

The aim of this study was to evaluate the association of the persistence of measurable C-peptide values with new glucose metrics obtained through interstitial glucose monitoring ( *i.e.* , glycemic variability) and the development of chronic microvascular and macrovascular complications in adult patients with T1DM.

## SUBJECTS AND METHODS TYPE OF STUDY AND PATIENT SELECTION

**Figure 1:**
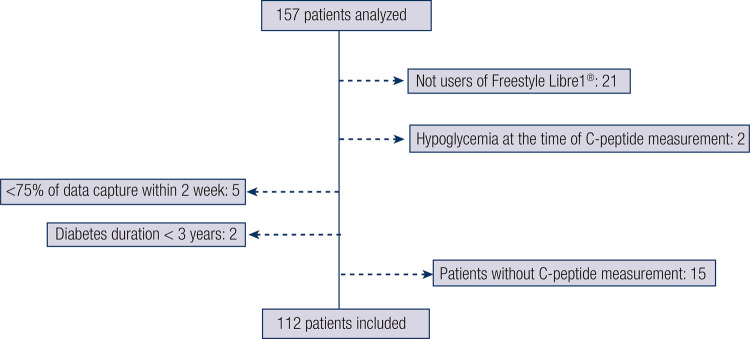
: Flowchart of the sample selection process.

This was a clinical research study with retrospective data collection from hospital medical records of patients following up at the Endocrinology Department of a tertiary center between 2019 and 2022. Adult patients with T1DM undergoing intensive insulin therapy through multiple daily injections (MDI) or continuous subcutaneous insulin infusion (CSII) were selected. For inclusion in the study, the patients had to meet the following inclusion criteria ( Figure 1 ):

- Diabetes duration > 3 years.
- Positive anti-GAD65, anti-IA2, and/or anti-ZnT8 antibodies.
- Plasma glucose ≥ 70 mg/dL concomitant with C-peptide measurement.
- ≥2 weeks of interstitial glucose monitoring using FreeStyle Libre 1 (Abbott Diabetes Care, Witney, UK) with ≥75% data captured prior to measurement of C-peptide level.

### Clinical data selection

Glucose metrics obtained through interstitial glucose monitoring were collected from the LibreView platform (Abbott) and included average glucose level (mg/dL), glucose standard deviation (SD; mg/dL), coefficient of variation (CV; %), time below range (TBR; %), time in range (TIR; %), time above range (TAR; %), and glucose management indicator (GMI; %).

Clinical and laboratory data were collected from the patients’ clinical records and included age (years), sex (female/male), body mass index (BMI; kg/m^2^), age at diagnosis (years), disease duration (years), physical exercise practice (yes/no), method of insulin administration (MDI/CSII), functional insulin treatment (yes/no), diabetes complications (diabetic kidney disease, retinopathy, neuropathy, stroke, myocardial infarction, and peripheral arterial disease; yes/no), the two most recent HbA1c measurements (%) spaced at least 3 months apart, and plasma C-peptide level (ng/mL) with concomitant glucose measurement (mg/dL).

Chronic vascular complications were diagnosed as follows:

- Retinopathy: presence of lesions detected by screening (retinography) and/or ophthalmological evaluation.
- Diabetic kidney disease: serum creatinine level corresponding to an estimated glomerular filtration rate < 60 mL/min/1.73 m^2^ and/or urinary albumin-to-creatinine ratio ≥ 30 mg/g (confirmed in at least two of three positive samples), both sustained for a period > 3 months.
- Neuropathy: absence of proprioceptive sensation (positive pinprick test) with or without other sensations (nociceptive, thermal, and vibratory), or history of a neuropathic foot ulcer.
- Macrovascular complications: evidence from medical records of established diagnosis of stroke, myocardial infarction, or peripheral arterial disease. In cases where macrovascular complications were not clearly reported in the medical records, we sought indirect evidence of these complications ( *e.g.* , in the case of myocardial infarction, areas of hypocontractility on echocardiography evidencing a previous coronary event).

### C-peptide assay

Blood for C-peptide level measurement was collected in the fasting state between 8 and 10 am. Patients with acute hyperglycemia or hypoglycemia at the time of blood collection were excluded to prevent these conditions from interfering with the C-peptide results. Additionally, those with C-peptide levels > 600 pmol/L (1.8 ng/mL) who were negative for all three autoantibodies were considered as probably not having T1DM and were excluded from the analysis.

Quantitative measurement of plasma C-peptide levels was obtained by sandwich immunoassay using Siemens Healthineers Atellica IM C-peptide (Siemens Healthcare GmbH, Erlangen, Germany). Results below the measurement range were reported as < 0.05 ng/mL, according to the manufacturer’s recommendation.

For comparative purposes, the included individuals were divided into two groups: undetectable C-peptide residual secretion (<0.05 ng/mL) and detectable C-peptide residual secretion (≥0.05 ng/mL).

### Statistical analysis

The data were analyzed using SPSS, version 27 (IBM Corp., Armonk, NY, USA). Descriptive statistics are presented as medians and quartiles (first quartile [Q1] – third quartile [Q3]) for quantitative variables and frequencies (n) and percentages (%) for categorical variables.

Univariate analysis for associations with detectable C-peptide was initially performed using the Mann-Whitney test for quantitative variables and the chi-square test for categorical variables. When Cochran’s rules were not met, the chi-square test was replaced by Fisher’s exact test.

The association between detectable C-peptide level and outcomes was assessed using linear regression for continuous outcomes and logistic regression for binary outcomes. For linear regression, the effect of a detectable C-peptide level on outcomes was estimated using unstandardized coefficients and their 95% confidence intervals. Coefficients estimation was based on the ordinary least squares method. For explained variance, we calculated R2 for unadjusted models and adjusted R2 for models that were adjusted. Assumptions and diagnostics of the linear regression model were checked. The normality of residuals was checked and confirmed with histograms and Kolmogorov-Smirnov tests. No outliers were found in the standardized, *i.e.* , all residuals were within the [-3, 3] interval. Homoscedasticity was checked by plotting standardized *versus* predicted values. The points were randomly distributed, showing no tendency, thus confirming this assumption. No evidence of multicollinearity was found, as the variance inflation factor values were close to 1 for all variables. In logistic regression, the effect of detectable C-peptide level on outcomes was estimated using odds ratios and 95% confidence intervals. The Hosmer-Lemeshow test was applied to assess the goodness of fit, with results showing p > 0.05 for all models. Model quality was assessed using Nagelkerke R2.

Models were implemented to analyze the crude effect of detectable C-peptide levels and the adjusted effect, controlling for sex, age at diagnosis, duration of diabetes, BMI, exercise, CSII, and functional insulin therapy.

P values < 0.05 were considered significant.

### Ethical considerations

The database was created considering the anonymity of the participants and was treated confidentially. Irreversible anonymization was performed at the time of data extraction. From that time, data were treated by the principal researcher with an individual numerical code for each participant. The data were maintained in a protected folder until the final publication of the study results. The study was conducted in accordance with ethical principles and good practice. The research protocol was approved by the Ethics Committee for Research in Life and Health Sciences of the Ethics Council of the University of Minho (CEICVS), the Data Protection Officer (DPO), and the Ethics Committee for Health of HB (CESHB).

## RESULTS

The final analysis included 112 adult patients with T1DM with a median age of 43 years (range 18–72 years, Q1: 31.5 – Q3: 56.5 years), of whom 65 (58.0%) were men. The median age at diagnosis was 22 years (Q1: 12 – Q3: 34 years), and the median diabetes duration was 18.5 years (Q1: 12 – Q3: 29 years). The median BMI was 24.4 kg/m^2^. Overall, 39.3% of the cohort practiced physical exercises. A total of 37.5% and 62.5% of the patients used CSII and MDI, respectively. Additionally, 63.1% of the cohort received functional insulin therapy.

The initial analysis did not identify significant associations between detectable C-peptide level and the various outcomes evaluated ( Table 1 ). However, regarding the various covariates evaluated, detectable C-peptide level was associated with older age at diagnosis (p = 0.002) and shorter diabetes duration (p = 0.004), while undetectable C-peptide level was associated with CSII use (p = 0.049).

**Table 1:** Descriptive statistics of outcomes and covariates stratified by C-peptide level

		C-peptide level		

	Total	Undetectable <0.05 ng/mL	Detectable >0.05 ng/mL	P value
**Outcomes**				
Average glucose (mg/dL)	170.0 (148.5-191.5)	170.5 (150.5-193.5)	169.5 (148.0-190.0)	p=0.273^a^
Glucose SD (mg/dL)	63.4 (51.8-79.6)	61.6 (51.6-85.1)	64.5 (52.1-74.3)	p=0.777^a^
Glucose CV (%)	39.2 (34.2-44.3)	39.6 (35.0-46.0)	38.1 (33.7-43.0)	p=0.297^a^
TBR (%)	3.0 (1.0-6.0)	4.0 (1.5-6.0)	2.0 (1.0-5.0)	p=0.149^a^
TIR (%)	55.0 (43.0-69.5)	51.5 (42.0-67.0)	59.5 (46.0-74.0)	p=0.081^a^
TAR (%)	40.0 (23.5-53.0)	41.5 (26.5-56.0)	39.5 (20.0-51.0)	p=0.188^a^
GMI (%)	7.4 (6.8-8.2)	7.4 (6.8-8.4)	7.4 (6.8-8.1)	p=0.420^a^
Average of the last two HbA1c measurements (%)	7.6 (7.0-8.2)	7.7 (6.9-8.2)	7.4 (7.0-8.0)	p=0.593^a^
Neuropathy (yes)	9 (8.0%)	7 (10.9%)	2 (4.2%)	p=0.296^c^
Nephropathy (yes)	20 (17.9%)	9 (14.1%)	11 (22.9%)	p=0.226^b^
Retinopathy (yes)	39 (34.8%)	26 (40.6%)	13 (27.1%)	p=0.137^b^
Macrovascular complications (yes)	5 (4.5%)	2 (3.1%)	3 (6.3%)	p=0.650^b^
**Covariates**				
Sex (male)	65 (58.0%)	35 (54.7%)	30 (62.5%)	p=0.407^b^
Sex (female)	47 (42.0%)	29 (45.3%)	18 (37.5%)	
Current age (years)	43.0(31.5-56.5)	42.5 (30.5-55.0)	44.5 (33.5-59.0)	p=0.443^a^
Age at diagnosis (years)	22.0 (12.0-34.0)	17.5 (9.0-28.0)	27.5 (16.5-38.5)	**p=0.002^a^**
Diabetes duration (years)	18.5 (12.0-29.0)	20.0 (14.0-32.0)	14.0 (9.0-24.0)	**p=0.004^a^**
BMI (kg/m^2^)	24.4 (22.6-28.2)	24.3 (22.0-28.6)	24.8 (23.0-28.2)	p=0.769^a^
Exercise (yes)	44 (39.3%)	22 (34.4%)	22 (45.8%)	p=0.219^b^
CSII (yes)	42 (37.5%)	29 (45.3%)	13 (27.1%)	**p=0.049^b^**
Functional insulin therapy (yes)	70 (63.1%)	38 (60.3%)	32 (66.7%)	p=0.492^b^

Results are presented as median (first quartile - third quartile) for quantitative variables and as π (%) for categorical variables. ^a^ Mann-Whitney test; ^b^ chi-square test; ^c^ Fisher's exact test. Abbreviations: **BMI,** body mass index; CSII, continuous subcutaneous insulin infusion; CV, coefficient of variation; **GMI,** glucose management indicator; **HbA1c,** glycated hemoglobin; **TBR,** time

below range; TAR, time above range; TIR, time in range.

Table 2 shows the effect of detectable C-peptide level on the outcomes. A single crude effect was observed in TIR (β = 7.13, p = 0.044), suggesting higher TIR in patients with detectable C-peptide level. This association was resistant to adjustment for confounders (aβ = 11.42, p = 0.001). Furthermore, after adjustment for covariates, several associations between detectable C-peptide level and outcomes were found that were not previously observed in the univariate analysis. Indeed, detectable C-peptide level was significantly associated with lower average glucose level (aβ = -14.48, p = 0.045), TAR (aβ = -11.03, p = 0.002), GMI (aβ = -0.55, p = 0.024), and HbA1c level (aβ = -0.41, p = 0.035). No significant associations were observed between detectable C-peptide level and glucose SD, glucose CV, TBR, and microvascular or macrovascular complications.

**Table 2:** Effect of detectable C-peptide level on various outcomes

		Detectable C-peptide effect (>0.05 ng/mL)				

	Unadjusted model			Adjusted model		

Outcome	β (p value)	95% CI	R^2^	aß (p value)	95% CI	aR^2^
Average glucose (mg/dL)	-6.99 (p=0.341)	-21.47; 7.50	0.00	**-14.48 (p=0.045)**	**-28.65; -0.32**	0.19
Glucose SD (mg/dL)	-0.52 (p=0.940)	-14.27; 13.24	0.00	-3.29 (p=00.632)	-17.03; 10.44	0.10
Glucose CV (%)	-2.05 (p=0.275)	-5.78; 1.68	0.02	-3.05 (p=00.121)	-6.93; 0.84	0.05
TBR (%)	-0.94 (p=0.316)	-2.28; 1.60	0.01	-0.34 (p=0.725)	-2.25; 1.57	0.09
TIR (%)	**7.13 (p=0.044)**	**0.19; 14.08**	0.04	**11.42 (p=0.001)**	**4.75; 18.08**	0.24
TAR (%)	-6.04 (p=0.106)	-13.39; 1.31	0.02	**-11.03 (p=0.002)**	**-18.06; -4.00**	0.24
GMI (%)	-0.29 (p=0.229)	-0.77; 0.19	0.01	**-0.55 (p=0.024)**	**-1.03; -0.08**	0.16
Average of the last two HbA1c (%) measurements	-0.22 (p=0.249)	-0.61; 0.16	0.01	**-0.41 (p=0.035)**	**-0.80; -0.03**	0.14
**Outcome**	**OR (p value)**	**95% CI**	**ngR^2^**	**aOR (p value)**	**95% CI**	**ngR^2^**
Neuropathy (yes)	0.35 (p=0.201)	0.07; 1.76	0.04	0.30 (p=0.297)	0.05; 2.45	0.49
Nephropathy (yes)	1.78 (p=0.245)	0.67; 4.73	0.02	2.92 (p=0.091)	0.84; 10.13	0.31
Retinopathy (yes)	0.53 (p=0.123)	0.24; 1.19	0.03	0.52 (p=0.233)	0.18; 1.52	0.48
Macrovascular complications (yes)	2.03 (p=0.447)	0.33; 12.68	0.02	3.76 (p=0.239)	0.41; 34.12	0.15

Model adjusted for sex, age at diagnosis, diabetes duration, body mass index, exercise, continuous subcutaneous insulin infusion, and functional insulin therapy. R2, R squared; aR2, adjusted R squared; ngR2, Nagelkerke R squared; β, linear regression coefficient; aß, adjusted linear regression coefficient. Abbreviations: aOR, adjusted odds ratio; CI, confidence interval; CV, coefficient of variation; GMI, glucose management indicator; HbA1c, glycated hemoglobin; TBR, time below range; TAR, time above range; TIR, time in range.

## DISCUSSION

In our sample of 112 patients with T1DM with a median disease duration of 18.5 (Q1: 12 – Q3: 29) years, 42.8% (n = 48) had detectable C-peptide level. This rate is lower than the one reported in the Joslin Medalist Study (in which disease duration was longer), although a direct comparison between this study and ours is limited due to variations in serum C-peptide sample collection conditions (random *versus* fasting, respectively) and the adoption of different C-peptide cutoff levels (0.012 ng/mL *versus* 0.05 ng/mL, respectively) ( 14 ).

We found that individuals with detectable C-peptide level were older than those with undetectable C-peptide level. The fact that the median age at diagnosis was also higher in the group with detectable C-peptide level probably indicates that this group had a greater proportion of patients with latent autoimmune diabetes of the adult (LADA), a subtype of T1DM characterized by a slower progression of autoimmune diabetes mellitus. This type of diabetes is associated with a slower decline in insulin production compared with T1DM and develops more frequently in individuals older than 30 years ( 15 , 16 ).

Notably, the median diabetes duration was shorter in the group with detectable C-peptide level, which is in line with previously published data. A progressive decline in C-peptide values is expected over the years until complete failure of insulin production is reached ( 17 , 18 ). In this sense – and in agreement with previously published data – we demonstrated that shorter disease duration and older age at diagnosis were significantly associated with detectable C-peptide level (p = 0.004 and p = 0.002, respectively) ( 14 , 17 , 18 ).

In our sample, a significantly larger proportion of patients with undetectable ( *versus* detectable) C-peptide level used CSII (45.3% *versus* 27.1%, respectively, p = 0.049). We consider that this finding is not related to the use of the device itself but to the fact that our national CSII program privileges CSII use during pediatric age; this finding suggests that the group with undetectable C-peptide level included patients who were younger at diagnosis and, consequently, had longer disease duration – two of the main factors determining residual C-peptide secretory capacity.

In patients with T1DM, preservation of beta-cell function, as measured by C-peptide level, is known to result in improved metabolic control. In our sample, after adjustments for covariates (sex, disease duration, BMI, and CSII use), we found that a detectable C-peptide level (≥0.05 ng/mL) was significantly associated with lower TAR (p = 0.002), TBR (p = 0.024), average glucose level (p = 0.0045), and HbA1c level (p = 0.035). Furthermore, patients with detectable C-peptide level had significantly higher TIR even before adjustment for covariates (unadjusted model: p = 0.044, adjusted model: p = 0.001). Considering these findings, we conclude that residual C-peptide secretion in adult patients with T1DM can indeed be considered a protective factor in the disease’s metabolic control.

Contrary to the findings by Gibb et al., we found no significant association between detectable C-peptide level and glycemic variability (CV) or TBR in our sample ( 13 ). This discrepancy can be partially explained by the fact that we had less available data related to the CV metric in the entire sample, so the association could not be demonstrated. Regarding TBR, although our patients with preserved C-peptide secretion had, on average, a lower percentage of TBR, the final analysis did not reach statistical significance. In addition, the lower accuracy of the FreeStyle Libre 1 system (the continuous glucose monitoring [CGM] system used by most of our patients) in detecting glucose levels in the hypoglycemic range and the lack of data about self-reported hypoglycemia may have contributed to this limitation ( 13 , 19 ).

Regarding the occurrence of chronic microvascular and macrovascular complications, some studies have concluded that patients with T1DM and higher fasting C-peptide values have a lower prevalence of microvascular complications, regardless of disease duration or average HbA1c value, while a similar association has not been found for macrovascular complications ( 20 , 21 ). As in other studies, our data did not confirm a significant relationship between C-peptide value and microvascular or macrovascular complications. This may have occurred because our sample was composed of young patients (median age 43 years) with short disease duration and acceptable glycemic control. Additionally, only a few patients presented complications ( *i.e.* , 9 patients with neuropathy, 20 patients with nephropathy, 39 patients with retinopathy, and 5 patients with macrovascular complications). We can also hypothesize that younger people have easier access to information related to the disease and may, thus, be more aware of measures and strategies to prevent complications. Although there was a lack of significant association between detectable C-peptide level and TBR, CV, or microvascular and macrovascular complications, careful observation of the results showed a trend toward lower CV and lower prevalence of diabetic kidney disease and macrovascular complications in the group with detectable C-peptide level.

A key strength of our study is its novelty in assessing the association between C-peptide level and new glucose metrics derived from CGM in a “real-world” clinical context. The association between CGM and C-peptide levels has only been analyzed in a single study that included a large pediatric population within 2 years from diagnosis and with relatively high C-peptide levels ( 22 ). As a “real-world” assessment, the various measures obtained in the present study (sociodemographic data, HbA1c and C-peptide levels, presence of complications, and flash monitoring data) were not captured simultaneously. However, we believe that this would increase the likelihood of a type II error rather than producing false-positive associations with C-peptide levels. Similar to other studies highlighting the concept of residual C-peptide secretion in patients with T1DM, we also found that – contrary to what has been previously postulated – not all patients with T1DM have absolute insulinopenia.

In terms of study limitations, it is important to highlight that some data were missing due to the retrospective design and real-world nature of the study. Additionally, flash glucose monitoring was used in several patients instead of the most accurate technology of real-time/CGM. It would also have been useful to report total insulin daily dose data, but this information was not consistently available in the patients’ medical records. Additionally, the C-peptide measurements analyzed were obtained in a fasting period. While formal stimulation tests ( *e.g.* , following intravenous glucagon or a standardized mixed-meal test) are most accurate and reproducible for research purposes, fasting or non-fasting (“random”) samples are usually suitable in clinical practice if the sampling conditions (timing relative to food and concomitant glucose measurement) are warranted ( 23 ). We defined 0.05 ng/mL as the cutoff value for C-peptide while considering the particularity of our laboratory assay, although it is recognized that the cutoff values for this measurement are not homogeneously defined in the literature. Acknowledging the dependence that we have on laboratory methodology, we opted to categorize the groups based on this cutoff value. Despite these limitations, we consider that the results of our study may open the door for further research and reinforce the need to link technology to better clinical outcomes for patients and medical teams.

In conclusion, in adult patients with T1DM, persistent C-peptide secretion is associated with significantly better metabolic control reflected by different glucose metrics obtained through CGM data, namely TIR, TAR, GMI, and HbA1c ( 24 ). Although we found no significant association between detectable C-peptide level and TBR, CV, or microvascular and macrovascular complications, we observed a positive trend in this respect. Based on these findings, persistent C-peptide secretion in adult patients with T1DM can be potentially considered an additional favorable factor in achieving glucose goals. Therefore, strategies to optimize C-peptide preservation across the lifespan in patients with T1DM should be offered during follow-up, not only through early improvement of glycemic control, but also through emerging therapeutic strategies, including the use of the anti-CD3 teplizumab or the calcium channel blocker verapamil ( 25 , 26 ).
